# Editorial: Advances in bioprocessing of viral vectors and virus-like particles

**DOI:** 10.3389/fbioe.2023.1166430

**Published:** 2023-03-14

**Authors:** Philipp Vormittag, Michael W. Wolff

**Affiliations:** ^1^ Lonza AG/Ltd, Drug Product Services, Basel, Switzerland; ^2^ Institute of Bioprocess Engineering and Pharmaceutical Technology, University of Applied Sciences Mittelhessen (THM), Giessen, Germany; ^3^ Bioresources, Fraunhofer Institute for Molecular Biology and Applied Ecology (IME), Giessen, Germany

**Keywords:** virus-like particles (VLP), vaccines, baculovirus expression vector system (BEVS), perfusion systems, membrane adsorber technique, downstream processing, upstream processing, viral vectors

Biological nanoplexes, such as viruses, virus-like particles (VLPs), phages, exosomes, and lipid nanoparticles are valuable tools in medicine. This is particularly the case for gene therapy to treat hereditary and acquired diseases, as well as for vaccines as prophylaxis. In fact, the latter are a growth engine for the biopharmaceutical industry, motivated by the continuous increase of infectious hazards, such as the recent COVID-19 pandemic. Vaccines are typically administered to healthy individuals and are, accordingly, subject to high safety requirements, which have to be considered in the manufacturing process. Biological nanoplexes are generally derived from living organisms and are more complex than traditional therapeutics in terms of their components and production technologies. This is equally true for vaccines based on encapsulated nucleic acids or recombinant proteins, which usually are structurally less complicated. Cell-based production processes for biological nanoplexes and recombinant proteins comprise three main segments: i) The upstream process (USP), in which the target components are manufactured, ii) the downstream process (DSP), where the components, produced in the USP, are purified in accordance with the regulatory requirements, and finally iii) the formulation and sterile filling of the formulated drug into appropriate primary packaging material.

Scientists from different fields, including virology and bioprocess engineering, continue to improve manufacturing processes to shorten their development timelines, and to meet the global demand on vaccines. These developments cannot compromise on product quality and purity, and should ideally drive the cost-effectiveness of the process. In order to meet these challenges, scientists focus on the following bioprocess and analytical Research Topic: i) In-process accelerated analytics (Lothert et al., 2022), ii) process automation and data science (Vormittag et al., 2020), and high-throughput technology to speed up process development (Marchel et al., 2019; Hillebrandt et al., 2021), iii) process translation from the development scale to the production scale, iv) platform technologies in the area of USP (Felberbaum, 2015; Farzaneh et al., 2017; Chambers et al., 2018; Tan et al., 2021) and DSP (Marichal-Gallardo et al., 2017; Fortuna et al., 2018; Hillebrandt et al., 2020; Lothert et al., 2020), v) continuous manufacturing processes (Krober et al., 2013; Fischer et al., 2018; Fortuna et al., 2021), vi) single-use technologies and closed systems, vii) modeling of production processes, and (viii) formulation strategies for nanoplexes (Batty et al., 2021). Some of this research is addressed in the following Research Topic.

For cell-derived vaccines and viral vectors, a wide range of cell substrates (e.g., microbial, fungal, insect and mammalian cell lines) is used for their production. The respective selection is largely driven by the required post-translational modification of the target components, by economic aspects, and by their suitability to be used as a platform technology for different viral targets. The application of prokaryotic cells is exemplified in this Research Topic by Valentic et al. for the production of hepatitis B core antigen (HBcAg)-based VLPs in *E. coli*. The study addresses VLPs as gene therapy vehicles, and investigates the influence of the HBcAg nucleic acid binding region on the loading capacity of the VLP constructs and their manufacturing process.

A commonly used eukaryotic system for the production of VLPs relies on the baculovirus expression vector system (BEVS). The system is addressed by Fernandes et al *.* For the making of a VLP influenza vaccine, composed of the influenza surface protein hemagglutinin (HA). In this context, they investigated the impact of cell retention devices, alternating tangential flow filtration (ATF) and tangential flow filtration (TFF), and the perfusion rate on cell growth (Sf-9 cells) and protein expression kinetics. Another article from Hong et al. describes the genetic engineering of the BEVS to improve protein production. Here, they provide a comprehensive overview of pharmaceutically relevant products produced by the BEVS, summarizing different insect cell lines and genetic approaches for the production of recombinant proteins. Furthermore, the engineering of insect cells is touched upon to modify, e.g., the glycosylation pathway of the host cells.

Despite all the developments in prokaryotic and simpler eukaryotic systems, it is frequently inevitable for the production of infectious virus particles to use mammalian or avian cells. In this regard, virus propagation is carried out over a wide range of cultivation processes with various cell lines such as, for example, Madin-Darby canine kidney cells, Madin-Darby bovine kidney cells, Vero cells (monkey kidney epithelial cell-derived), PER.C6 (human fetal retinoblast cell-derived), EB66 (duck embryonic stem cell-derived), AGE1.CR (duck embryonic cell-derived), and human embryonic kidney 293 cells (HEK-293). Currently, variants of the latter are frequently used for the production of viral particles and are addressed in three articles of this Research Topic. In this context, Tran and Kamen investigated the production of lentiviral vectors in HEK-293 cells in a perfusion process (3L bioreactor scale), using tangential flow depth filtration (TFDF) for cell retention, and compared this with a small scale shake flask approach. Another article by Mendes et al. focused on the comparison of ATF and TFDF techniques for cell retention in a perfusion bioreactor to produce adeno-associated virus 8 (AAV8) in HEK-293 cells.

The DSP for biopharmaceutical products involves a series of individual unit operations for clarification, primary and secondary purification, and polishing. For many biotechnological products, including vaccines and viral vectors, the DSP is still the bottleneck in the manufacturing processes. The second area addressed in the study by Mendes et al. goes beyond classical filtration applications of biopharmaceutical processes. Traditionally, filtration techniques are used to remove particular process contaminants, such as cell debris, product concentration, buffer exchange, and sterile filtration. Mendes et al. investigated the suitability of ATF and TFDF to combine the AAV harvest and clarification into one single process step. Similar topics are addressed in other studies with the same objective, to intensify production processes for biological nanoplexes. In these research projects, variations of TFF are combined with precipitation, re-dissolution, and multimodal size-exclusion chromatography (Hillebrandt et al., 2020; Hillebrandt et al., 2021).

The third publication, using HEK-293 cells for virus production contributed by Gautam et al., centers on a purification method commonly used further downstream in the process. They investigated the purification of a pseudo-typed oncolytic vesicular stomatitis virus by a cation-exchange membrane adsorber chromatography. Chromatography is a commonly used process unit in the DSP of biological nanoplexes, for which usually convective matrices are applied. The main advantage, compared to conventional resins, is the flow-independent performance over a wide range of flow regimes. Furthermore, in the case of bead-based resins, larger particles and macromolecules are commonly limited by pore diffusion or pore exclusion effects. For this reason, the matrices for larger products generally consist of membranes, as in the presented example from Gautam et al., as well as nanofibers, or monoliths. Alternatively, if the process contaminants are to be adsorbed, restricted access matrices are widely used. These chromatographic stationary phases are designed to capture low molecular weight contaminants and, concomitantly, exclude larger particles and macromolecules.

The work presented in this Research Topic represents the ongoing efforts to optimize and accelerate the production process of biological nanoplexes and vaccines, which will pave the way for upcoming developments to overcome the current challenges in vaccine production.
